# Byron’s Last Moments

**Published:** 1884-07

**Authors:** 


### BYRON'S LAST MOMENTS.

THE last illness and death of Byron are not less curious to the medical mind than the life of the man. In brief, Byron went to Greece in his latter days (he was only 36,) ostensibly to liberate Greece, actually to obtain the crown of a kingdom he had hoped to see established—a crown which he did, in fact, nearly secure. His death by epileptic seizures and by exposure to malaria, was clinched, it is generally felt, by medical perseverance in crystallized error. Two "youthful and incompetent" doctors, to quote Jefferson's definition of them, Bruno and Millingen, "did their best and their worst" for him. He had been living, by his own rule, for five weeks on toast and tea, and at last, in response to the urgent appeal and insistance of the doctors, he consented to be bled (date, April 19, 1824). Casting at the two the fiercest glance of vexation, and throwing out his arm, he said in his angriest tone: "There! You are, I see, a —— set of butchers! Take away as much blood as you like, and have done with it." They took twenty ounces. The next day they repeated the bleeding twice, and put blisters above the knee, because he objected to having his feet exposed for the blistering process. In spite of all he lived on, and on the 18th actually rose from his bed and tottered into an adjoining room, leaning on his servant Tita's arm. There he amused himself with a book for a few minutes, and then returned to bed. In the afternoon two new and strange doctors came to look at him, and after they had left he took one anodyne draught. Some time later he took another draught of a similar kind. and at 6 o'clock he uttered his last intelligible sentence: "Now I shall go to sleep." He slept for twentyfour hours, and at fifteen minutes past six on the evening of April 19, surprised his watchers by opening his eyes and instantly shutting them. "He died at that instant." In this day we look with wonder at the medical art which in twenty four hours could bleed three times a fasting man, then blister him, and finally supplement the so-called treatment with two strong narcotic draughts.

